# Supplementary material for: The Systems Biology Research Tool: evolvable open-source software
Source: BMC Syst Biol. 2008 Jun 29;2:55. doi: 10.1186/1752-0509-2-55 (PMC2446383; doi:10.1186/1752-0509-2-55)
Supplement: Additional file 1 — SBRT Archive. An archive of the current version of the Systems Biology Research Tool. [file 1752-0509-2-55-S1.zip › sbrt-1.4.0/doc/developers_guide/api/sbrt/kernel/util/class-use/UnmodifiableMap.html]

Uses of Class sbrt.kernel.util.UnmodifiableMap


|  |  |  |
| --- | --- | --- |
| **Overview** \| **Package** \| **Class** \| **Use** \| **Tree** \| **Deprecated** \| **Index** \| **Help** | |  |
| PREV   NEXT | **FRAMES**    **NO FRAMES**     **All Classes** |  |


---


## **Uses of Class sbrt.kernel.util.UnmodifiableMap**

| Packages that use UnmodifiableMap | |
| --- | --- |
| **sbrt.ext.cplex** | Provides a class that allows communication with CPLEX. |
| **sbrt.kernel.fba** | Provides classes and interfaces for creating objects relevant to Flux Balance Analysis. |
| **sbrt.kernel.fba.comp** | Provides classes and interfaces for performing Flux Balance Analysis computations. |
| **sbrt.kernel.math** | Provides classes and interfaces for creating objects relevant to mathematics. |
| **sbrt.kernel.math.geom.comp** | Provides classes and interfaces for performing geometry-related computations. |
| **sbrt.shell** | Provides classes used by shell-level software. |
| **sbrt.shell.io** | Provides classes and interfaces for reading and writing files. |
| **sbrt.shell.io.fba** | Provides classes and interfaces for reading and writing files relevant to Flux Balance Analysis. |
| **sbrt.shell.mng.fba** | Provides classes and interfaces for creating Flux Balance Analysis process managers. |
| **sbrt.shell.mng.stat** | Provides classes and interfaces for creating statistical process managers. |

Uses of UnmodifiableMap in sbrt.ext.cplex

| Methods in sbrt.ext.cplex that return UnmodifiableMap | |
| --- | --- |
| `UnmodifiableMap<C,java.lang.Double>` | `CplexSolver.getSolutionVector()`             Returns the most recently computed solution vector. |

Uses of UnmodifiableMap in sbrt.kernel.fba

| Methods in sbrt.kernel.fba that return UnmodifiableMap | |
| --- | --- |
| `UnmodifiableMap<MathExpr<java.lang.String>,Interval>` | `FluxConstraints.getExprConstraints()`             Returns the constraints for mathematical expressions of fluxes. |
| `UnmodifiableMap<java.lang.String,Interval>` | `FluxConstraints.getFluxConstraints()`             Returns the flux constraints for single reactions. |

Uses of UnmodifiableMap in sbrt.kernel.fba.comp

| Methods in sbrt.kernel.fba.comp that return UnmodifiableMap | |
| --- | --- |
| `UnmodifiableMap<java.lang.String,java.lang.Double>` | `InvalidFluxVectorException.getFluxVector()`             Returns the invalid flux vector; |

Uses of UnmodifiableMap in sbrt.kernel.math

| Methods in sbrt.kernel.math that return UnmodifiableMap | |
| --- | --- |
| `UnmodifiableMap<?,?>` | `InvalidPointException.getPoint()`             Returns the invalid point. |

Uses of UnmodifiableMap in sbrt.kernel.math.geom.comp

| Methods in sbrt.kernel.math.geom.comp that return UnmodifiableMap | |
| --- | --- |
| `UnmodifiableMap<V,java.lang.Double>` | `CdHarComputer.Results.getPoint()`             Returns the random interior point that was computed. |

Uses of UnmodifiableMap in sbrt.shell

| Fields in sbrt.shell declared as UnmodifiableMap | |
| --- | --- |
| `static UnmodifiableMap<java.lang.String,java.lang.String>` | `ProgramSolverFactory.nameClassMap`             The correspondence between the strings used to denote a particular program solver and the name of the actual class it represents. |

Uses of UnmodifiableMap in sbrt.shell.io

| Methods in sbrt.shell.io that return UnmodifiableMap | |
| --- | --- |
| `UnmodifiableMap<K,V>` | `RomiFile.getMap()`             Returns the map contained in this file. |

Uses of UnmodifiableMap in sbrt.shell.io.fba

| Methods in sbrt.shell.io.fba that return UnmodifiableMap | |
| --- | --- |
| `UnmodifiableMap<java.lang.String,java.util.Set<LinearComb<java.lang.String>>>` | `FluxCapInputFile.getFluxCaps()`             Returns the flux caps contained in this file. |

Uses of UnmodifiableMap in sbrt.shell.mng.fba

| Methods in sbrt.shell.mng.fba that return UnmodifiableMap | |
| --- | --- |
| `static UnmodifiableMap<java.lang.String,java.util.Set<LinearComb<java.lang.String>>>` | `MsFluxIntervalManager.getFluxCaps(ManagerInput<java.lang.String,java.lang.String> input, java.io.PrintWriter out, Fluxome fluxome)`             Returns the flux caps contained in the file with the name specified by `MsFluxIntervalManager.FLUX_CAP_FILE` in the provided input. |

Uses of UnmodifiableMap in sbrt.shell.mng.stat

| Methods in sbrt.shell.mng.stat that return UnmodifiableMap | |
| --- | --- |
| `static UnmodifiableMap<java.lang.String,java.lang.Double>` | `CorrelationManager.getVector(java.lang.String fileName)`             Parses the file with the provided name and returns the vector it contains. |
| `static UnmodifiableMap<java.lang.String,java.lang.Double>` | `CorrelationManager.getVector(java.lang.String fileName, java.io.PrintWriter out)`             Parses the file with the provided name, prints a status message to the provided print writer, and returns the vector the file contains. |
| `static UnmodifiableMap<java.lang.String,java.lang.Double>` | `CorrelationManager.getXValues(ManagerInput<java.lang.String,java.lang.String> input, java.io.PrintWriter out)`             Returns the parsed value for `CorrelationManager.X_FILE_NAME` in the provided input and prints a descriptive message to the provided print writer. |
| `static UnmodifiableMap<java.lang.String,java.lang.Double>` | `CorrelationManager.getYValues(ManagerInput<java.lang.String,java.lang.String> input, java.io.PrintWriter out)`             Returns the parsed value for `CorrelationManager.Y_FILE_NAME` in the provided input and prints a descriptive message to the provided print writer. |

---


|  |  |  |
| --- | --- | --- |
| **Overview** \| **Package** \| **Class** \| **Use** \| **Tree** \| **Deprecated** \| **Index** \| **Help** | |  |
| PREV   NEXT | **FRAMES**    **NO FRAMES**     **All Classes** |  |


---
